# The responsiveness of the PROMIS instruments and the qDASH in an upper extremity population

**DOI:** 10.1186/s41687-017-0019-0

**Published:** 2017-11-28

**Authors:** Man Hung, Charles L. Saltzman, Tom Greene, Maren W. Voss, Jerry Bounsanga, Yushan Gu, Angela A. Wang, Douglas Hutchinson, Andrew R. Tyser

**Affiliations:** 1 Department of Orthopaedic Surgery Operations, University of Utah, School of Medicine, 590 Wakara Way, Salt Lake City, UT 84108 USA; 2 Division of Public Health, University of Utah, School of Medicine, 375 Chipeta Way Ste. A, Salt Lake City, 84108 USA; 3 Population Health Foundation, University of Utah, 295 Chipeta Way, Williams Building, Room 1C448, Salt Lake City, UT 84132 USA

**Keywords:** Responsiveness, Patient-reported outcomes, PROMIS, qDASH, Physical function, Pain, Orthopaedics

## Abstract

**Background:**

This study evaluated the responsiveness of several PROMIS patient-reported outcome measures in patients with hand and upper extremity disorders and provided comparisons with the qDASH instrument.

**Methods:**

The PROMIS Upper Extremity computer adaptive test (UE CAT) v1.2, the PROMIS Physical Function (PF) CAT v1.2, the PROMIS Pain Interference (PI) CAT v1.1 and the qDASH were administered to patients presenting to an orthopaedic hand clinic during the years 2014–2016, along with anchor questions. The responsiveness of these instruments was assessed using anchor based methods. Changes in functional outcomes were evaluated by paired-sample t-test, effect size, and standardized response mean.

**Results:**

There were a total of 255 patients (131 females and 124 males) with an average age of 50.75 years (SD = 15.84) included in our study. Based on the change and no change scores, there were three instances (PI at 3 months, PI >3 months, and qDASH >3 months follow-ups) where scores differed between those experiencing clinically meaningful change versus no clinically meaningful change. Effect sizes for the responsiveness of all instruments were large and ranged from 0.80–1.48. All four instruments demonstrated high responsiveness, with a standardized response mean ranging from 1.05 to 1.63.

**Conclusion:**

The PROMIS UE CAT, PF CAT, PI CAT, and qDASH are responsive to patient-reported functional change in the hand and upper extremity patient population.

## Background

There has been an important shift toward the use and development of quality patient-reported outcome (PRO) instruments that minimize responder burden and exhibit sufficient reliability, validity, and clinical relevance. [1] These tools can assist in the accurate measurement of clinical outcomes, which are fundamental for rigorous clinical research as well as in improving the quality of care offered to patients. In order for PRO instruments to have these desired research and clinical benefits, validation studies are critical. Fitting this new emphasis, the Journal of Patient-Reported Outcomes has included rigorous studies on the development and evaluation of PROs in the aims and scope of the journal. [2] Determining whether a PRO instrument is responsive—i.e. able to detect a change in a patient’s reported health status or function—is an important pre-requisite for using such instruments to assess treatment effect.

The Patient-Reported Outcomes Measurement Information System (PROMIS) health measure improvement initiative was funded by the National Institutes of Health with the purpose of improving the quality of PROs. The development took a systematic approach, drawing from instruments already in use. Existing items were categorized, reviewed, and revised, creating a large pool of items that were then validated with item-response theory to allow for computer adapted testing, making the PROMIS instruments an important contribution to clinical and research practice while minimizing respondent burden. [3–5] The PROMIS Physical Function Computer Adaptive Test (PF CAT) and PROMIS Upper Extremity (UE) CAT instruments can be utilized to measure patients’ self-reported upper extremity health status, and have several advantages over other metrics. [6] The PROMIS UE and PF CAT have both demonstrated favorable performance characteristics and correlate well with the shortened version of the Disabilities of the Arm, Shoulder, and Hand (qDASH) in an orthopaedic upper extremity patient population. [7, 8] The responsiveness of these PROMIS instruments have not yet been evaluated in this same patient population.

Assessing responsiveness requires longitudinal data with repeated measures, where the same individual is assessed with the same instrument on at least two occasions. [9] Responsiveness can be assessed with either internal or external methods. Internal analysis of responsiveness evaluates the level of change based on the size of the differences between scores, and how much scores vary over time. [10] External responsiveness methods use an external anchor to relate the level of change to some other meaningful report of patient change, either a clinical gold-standard assessment or the patient’s own report of change. [11, 12] Detecting change is particularly important for PRO instruments if they are to be used to guide decisions in clinical practice.

The purpose of this study, therefore, is to evaluate the responsiveness of three PROMIS patient-reported outcome measures in patients with hand and upper extremity (non-shoulder) disorders and provide comparisons with the qDASH legacy instrument.

## Methods

### Patient sample

Institutional Review Board approval was obtained prior to the start of this study and informed consent was obtained from all participants as they sought medical care for orthopaedic conditions. The sample consisted of 255 new patients presenting to an academic upper-extremity (non-shoulder) clinic between the years of 2014 and 2016. All patients were 18 years or older and sought treatment for upper extremity musculoskeletal conditions. At the time of their clinic visits and prior to seeing a physician, patients were administered anchor questions and PROs electronically on a handheld tablet computer. Patients were recruited consecutively and PROs were administered as part of the standard clinic treatment protocol, with 1.5% of patients refusing to participate clinic-wide.

Patients were seen for a variety of upper extremity conditions with treatments including wound and bone care, skin grafts, tendon/ligament repair, incisions, implants, bursas, reconstructions, fractures, transplants, decompression, arthroscopy, endoscopy, nerve blocks, and carpel tunnel surgery. Depending on individual patient circumstance and timing in follow-up care, different patient samples could be included in the different follow-up periods (see Table 1). Also, depending on the diagnostic condition and treatment planning, patients differed in the amount or type of treatment received during the follow-up periods. This variation in treatment and follow-up timing is typical of a standard UE orthopaedic practice. Four patient follow-up periods were examined in this study: (1) 3-month follow-up (i.e., 80 to 100 days after initial assessment; (2) >3-month follow-up (i.e., 90 days or more after initial assessment); (3) 6-month follow-up (i.e., 170 to 190 days after initial assessment); and (4) >6-month follow-up (i.e., 180 days or more after initial assessment). Three and six-months are common time-periods for follow-up in orthopaedic practice. [13–20] These time-points were included in this analysis to correspond with prior literature and clinical practice.

**Table 1:** Demographics of patients

Patient characteristics	*n*	Percent	Mean (SD)	Range
Age (years)			50.75 (15.84)	18–90
Gender				
Male	124	48.6		
Female	131	51.4		
Race				
White or Caucasian	221	86.7		
Asian	4	1.6		
American Indian and Alaska Native	3	1.2		
Black or African American	6	2.4		
Other	15	5.9		
Missing	6	2.4		
Ethnicity				
Hispanic	18	7.1		
Non-Hispanic	232	91.0		
Missing	5	1.9		
Tobacco User				
Yes	25	9.8		
No	211	82.7		
Missing	19	7.5		
Procedure Type				
Removal of implant	4	1.6		
Excision, repair, surgery on the Humerus	7	2.8		
Excision, repair, surgery on the wrist or forearm	17	6.7		
Excision, repair, surgery on the hands and fingers	43	16.8		
Amputation procedures on the hand	1	0.4		
Neuroplasty, neurorrhaphy, arthroscopy, and misc. procedures	133	52.2		
Missing	50	19.5		
Insurance Provider				
Industrial/Workers Compensation	23	9.0		
Medicaid	1	0.4		
Medicare	49	19.2		
No Fault Auto Insurance	3	1.2		
Private Insurance	168	66.2		
Self-Pay	6	2.4		
Tricare	3	1.2		
Employment Status				
Disabled	14	5.5		
Full Time	121	47.5		
Part Time	13	5.1		
Not employed	28	11.0		
Retired	36	14.1		
Self Employed	13	5.1		
Student	9	3.5		
Missing	21	8.2		

### Patient-reported outcome measures

Three PROMIS instruments were administered to the patients: the PROMIS UE CATv1.2, the PROMIS PF CAT v1.2, and the PROMIS Pain Interference (PI) CAT v1.1. The PROMIS PF CAT v1.2 contains both upper extremity and lower extremity items and draws from a 121-item test bank. The PROMIS UE CAT v1.2 has a 16-item test bank and the PROMIS PI CAT v1.1 has a 40-item test bank. The qDASH was also administered, which is an 11-item, validated, shortened version of the 30-item Disabilities of the Arm and Shoulder (DASH) instrument. [21] The PROMIS instruments were made available through the Assessment Center, a secure web-based portal established by PROMIS developers. [22] Each of the four instruments were administered at baseline (i.e., either within seven days prior to the clinic visit of a new upper extremity condition or on the day of the first clinic visit) and at each follow-up visit patients attended.

All PROMIS instruments were calibrated in the general population with a mean of 50 and a standard deviation of 10 in the T-score scale, with patient scores primarily clustering between 20 to 80 points. [23] The larger the PROMIS PF or UE scores, the higher were the patients’ function, where the larger the PROMIS PI scores, the greater the pain interference experienced by the patients. The qDASH scores ranged from 0 to 100 with higher scores representing lower functioning levels.

### Anchor questions

For physical function, patient responses were anchored by the question; ‘Compared to your FIRST EVALUATION at the University Orthopaedic Center: how would you describe your physical function now?’(much worse, worse, slightly worse, no change, slightly improved, improved, much improved). The idea of anchoring a change score to some other measure of patient outcome is to provide a reference point. When that reference point comes from patient reports of noticeable improvement or decline, it may be considered a meaningful level of change. [24] Patients reporting meaningful change (much worse, worse, improved, much improved) were included in the responsiveness analysis to detect the ability of the PROs to measure meaningful levels of change. [25] When there is symmetry in data characteristic, the improved and deteriorated change groups can be considered together creating a distinction between those experiencing change versus those with stable symptomology. [26]

For the PI, the anchor question queried pain (i.e., Compared to your FIRST EVALUATION at the University Orthopaedic Center: how would you describe your episodes of PAIN now?) rather than physical function, and patients reporting pain which was worse, much worse, improved, or much improved since their first clinic visit were included in the responsiveness analyses.

### Statistical analysis

Patient demographics were examined and changes in their functional and pain outcomes were evaluated at four time points. Baseline scores were compared to the three-month follow-up scores (90 days plus or minus 10 days), six-month follow-up scores (180 days plus or minus 10 days), 90 days and beyond follow-up scores, and 180 days and beyond follow-up scores on all four patient-reported measures.

Change in the PRO metrics was calculated as the absolute value difference between the baseline score and the follow-up score for each patient. A paired sample two sided t-test was used to test the hypothesis that there was no difference in the PRO measures between time points on an individual patient level [10], with significance level set at *p* = 0.05. ANOVA was run to test the hypothesis that patients did not differ across levels of change.

A standardized measure of effect size (ES) was calculated using the Cohen’s d. Cohen’s d computes the difference in score between the baseline and the follow-up and then divides this difference by the baseline score standard deviation. This method takes into consideration the variability in scores, a step beyond the mean differences considered in the paired sample t-test [10]. In interpreting Cohen’s d, a small, medium, and large ES can be considered as d = 0.20, 0.50, and 0.80 respectively.

The standardized response mean (SRM) is another important indicator of ES, similar to the paired t-test, but removing dependence on sample size from the equation. [10] This is computed as the mean difference between baseline and follow-up PRO scores divided by the standard deviation of difference scores, reflecting individual changes in scores. Although there is not perfect consensus, recommended guidelines for interpreting SRM values are similar to interpretation of Cohen’s d. [10] All analyses were performed using either SPSS 23.0 (IBM SPSS Statistics for Windows, Armonk, NY: IBM Corp.), [27] or R 3.30 (R Development Core Team, Vienna, AT: R Foundation for Statistical Computing,) [28].

## Results

This study included a total of 131 females and 124 males with ages ranging from 18 years to 90 years (mean age = 50.75, SD = 15.84). For demographic information including gender, race, ethnicity, tobacco use, procedure and insurance type, see Table 1.

Mean, SD, range, and median along with mean differences of scores of the PROMIS UE, PF, PI and qDASH are presented in Table 2. Mean change scores for the PROMIS PI ranged from 4.81–10.68 whereas mean for no change scores ranged from 4.32–6.05. The PROMIS PI at 3-month and >3-month follow-up and the qDASH at >3-month follow-up were the only measures and only time-points with confidence intervals (CI)‘s showing a substantial difference between change groups (see Table 3). The PROMIS PF mean change scores ranged from 8.36–8.91 whereas mean for no change scores ranged from 5.92–9.00. The UE had mean change scores ranging from 7.57–9.51 and mean no change scores ranging from 6.67–8.21. Lastly, the qDASH showed mean change scores between 18.18 and 24.22 and mean no change scores between 17.21 and 24.40.

**Table 2:** Descriptive statistics of PROMIS instruments and qDASH of patients

Instrument	*n*	Mean (SD)	Range	Median
Baseline				
PROMIS PF	254	45.45 (9.53)	23.47–70.25	43.18
PROMIS PI	254	60.85 (7.34)	38.67–80.96	61.52
PROMIS UE	254	32.48 (9.28)	14.66–56.39	32.13
qDASH	255	50.09 (22.53)	4.54–97.73	50.00
3-month follow-up				
PROMIS PF	31	50.61 (10.73)	33.10–50.61	51.45
PROMIS PI	31	52.77 (8.62)	38.67–71.60	52.57
PROMIS UE	28	36.89 (10.14)	18.34–56.39	36.53
qDASH	29	33.39 (23.74)	2.27–79.54	27.27
>3-month follow-up				
PROMIS PF	151	46.34 (8.76)	24.07–73.28	47.41
PROMIS PI	177	56.20 (8.47)	38.67–80.07	55.98
PROMIS UE	148	34.95 (8.30)	18.34–56.39	35.09
qDASH	149	39.73 (22.76)	2.27–97.72	36.36
6-month follow-up				
PROMIS PF	18	47.70 (5.59)	32.60–56.06	47.83
PROMIS PI	20	55.94 (3.46)	50.12–62.64	55.10
PROMIS UE	18	35.62 (8.13)	18.35–56.26	35.85
qDASH	18	34.84 (16.16)	11.36–77.27	35.23
>6-month follow-up				
PROMIS PF	53	44.51 (10.23)	2.84–70.25	44.72
PROMIS PI	62	56.77 (8.87)	38.67–79.99	55.98
PROMIS UE	52	33.77 (8.66)	17.74–56.39	34.54
qDASH	55	41.79 (25.67)	6.82–93.18	36.36

**Table 3:** Mean Score Changes for PROMIS Instruments and qDASH

Instrument	*n*	No Change (SD)	*n*	Change (SD)	Mean Difference [95% CI]^a^
3-month follow-up					
PROMIS PF	29	9.00 (8.18)	31	8.64 (8.20)	0.36 [−3.88, 4.59]
PROMIS PI	25	5.95 (7.51)	31	10.68 (6.56)	−1.47 [−8.50, −0.96]
PROMIS UE	28	8.04 (6.19)	28	9.51 (7.54)	0.18 [−5.17, 2.24]
qDASH	30	24.40 (20.53)	29	24.22 (16.81)	−4.72 [−9.62, 9.98]
>3-month follow-up					
PROMIS PF	177	7.14 (6.85)	151	8.53 (7.31)	−1.39 [−2.93, 0.15]
PROMIS PI	145	6.05 (5.78)	177	7.48 (6.86)	−1.44 [−2.82, −0.05]
PROMIS UE	173	7.44 (6.46)	148	8.54 (6.86)	−1.10 [−2.56, 0.36]
qDASH	175	18.23 (17.10)	149	22.34 (17.75)	−4.10 [−7.93, −0.27]
6-month follow-up					
PROMIS PF	11	5.92 (6.23)	18	8.91 (7.06)	−2.99 [−8.30, 2.33]
PROMIS PI	9	4.32 (3.70)	20	4.81 (4.16)	−0.48 [−3.80, 2.83]
PROMIS UE	11	8.21 (5.46)	18	7.57 (5.33)	0.64 [−3.58, 4.87]
qDASH	11	17.77 (14.40)	18	18.18 (13.34)	−0.41 [−11.60, 10.77]
>6-month follow-up					
PROMIS PF	78	6.73 (5.65)	53	8.36 (6.67)	−1.62 [−3.78, 0.53]
PROMIS PI	69	5.97 (5.35)	62	6.71 (5.85)	−0.74 [−2.69, 1.20]
PROMIS UE	76	6.67 (6.50)	52	8.37 (5.84)	−1.70 [−3.92, 0.52]
qDASH	81	17.21 (17.09)	55	21.86 (17.34)	−4.65 [−10.60, 1.29]

^a^This is the mean difference with its associated 95% confidence interval between the no change score and the change score

Only 20% of the patient sample had baseline PROMIS PF scores at the average 50th percentile T-score of 50, 5% had PROMIS UE scores over 50, and 5% had an average PROMIS PI pain score of 50, indicating this group had low levels of function and high levels of pain at baseline.

### Paired t-test

At the 3-month, 6-month and >3-month follow-up, changes from baseline scores were significant for all instruments (*p* < 0.05). However, score changes for the >6-month time period varied in significance. The only instrument that did not show a significant change in scores was the UE CAT (*p* = 0.253), whereas the PF CAT, PI CAT, and qDASH showed significant changes in scores (*p* < 0.05; see Table 4). For all instruments, the baseline scores were not significantly different between the patients with missing and non-missing follow-up visit scores at all time points (*p* < 0.05) (results available upon request).

**Table 4:** Responsiveness of PROMIS instruments and qDASH of patients from baseline

Follow-up Period	Instrument	*n*	SRM [95% CI]	ES [95% CI] *p*-value	Paired t-test
3-month follow-up					
	PROMIS PF	31	1.05 [0.51, 1.57]	0.84 [0.31, 1.35]	< 0.001
	PROMIS PI	31	1.63 [1.04, 2.18]	1.48 [0.90, 2.02]	< 0.001
	PROMIS UE	28	1.26 [0.67, 1.81]	1.05 [0.48, 1.59]	0.006
	qDASH	29	1.44 [0.84, 2.00]	1.12 [0.28, 1.66]	< 0.001
>3-month follow-up					
	PROMIS PF	150	1.16 [0.91, 1.40]	0.92 [0.68, 1.16]	< 0.001
	PROMIS PI	176	1.09 [0.86, 1.31]	0.99 [0.77, 1.21]	< 0.001
	PROMIS UE	148	1.24 [0.99, 1.49]	0.88 [0.64, 1.12]	0.001
	qDASH	149	1.26 [1.01, 1.51]	0.97 [0.73, 1.21]	< 0.001
6-month follow-up					
	PROMIS PF	18	1.26 [0.52, 1.94]	0.83 [0.13, 1.49]	< 0.001
	PROMIS PI	20	1.16 [0.47, 1.80]	0.79 [0.13, 1.42]	< 0.001
	PROMIS UE	18	1.42 [0.66, 2.12]	0.85 [0.15, 1.51]	0.001
	qDASH	18	1.36 [0.61, 2.05]	0.80 [0.10, 1.46]	< 0.001
>6-month follow-up					
	PROMIS PF	52	1.25 [0.82, 1.66]	0.87 [0.46, 1.27]	0.033
	PROMIS PI	61	1.15 [0.76, 1.53]	0.96 [0.58, 1.33]	< 0.001
	PROMIS UE	52	1.43 [0.99, 1.85]	0.85 [0.44, 1.24]	0.253
	qDASH	55	1.26 [0.84, 1.66]	0.93 [0.53, 1.32]	0.006

### Effect size

All four instruments showed a high degree of responsiveness across all four follow-up periods. For the 3-month follow-up group, all instruments had high responsiveness ranging from 0.84–1.48. The instrument that was the most responsive for the 3-month follow-up was the PI CAT (1.48), whereas the PF CAT was the least responsive (0.84).

The 6-month follow-up also showed high responsiveness ranging from 0.79–0.85. The PI CAT was the least responsive at the 6-month follow-up (0.79) whereas the UE CAT was the most responsive (0.85). When looking at the >3-month follow-up time period of 90 days or more, responsiveness was still high (0.92–0.99). The least responsive measurement for this time period was the UE CAT (0.92) while the PI CAT showed the highest responsiveness (0.99). For the >6-month time period of 180 days or more, all instruments still showed high responsiveness but the PI CAT was the most responsive (0.97) whereas the UE CAT was the least (0.85). Overall, the PI CAT was consistently the most responsive to change when looking at ES (see Table 4). The 95% CI’s of the effect sizes demonstrates a meaningful difference in measure responsiveness at each follow-up time-point for each instrument, though the CI range for all measures dipped to include potential for a small effect in the 6-month follow-up period.

### Standardized response mean

All instruments had high responsiveness as measured by the SRM (1.05–1.63). The 95% CI’s around the SRM were all medium-large, ranging from 0.51–2.18, and reflect the overall larger size of effect as measured by the SRM compared to the ES on every measure at every time-point. In the 3-month follow-up group, the most responsive instrument was the PI CAT (1.63) while the PF CAT was the least responsive instrument (1.05) among the four. The 6-month follow-up showed that the PROMIS UE was the most responsive (1.42) whereas the PI CAT was the least (1.16). In the >3-month follow-up time period of 90 days or more, the PI CAT remained the least responsive instrument (1.09) whereas the qDASH was the most responsive (1.26). However, the UE CAT had the highest SRM (1.43) while the PI CAT had the lowest (1.15) for the >6-month follow up time period of 180 days or more. In general, the UE CAT was the most responsive to change when applying the SRM (see Table 4).

## Discussion

The main finding of this study is that the PROMIS Upper Extremity CAT, Physical Function CAT, and Pain Interference CAT are responsive to patient reported functional change in a hand and upper extremity (non-shoulder) orthopaedic population. In addition, the magnitude of the responsiveness for each instrument was large. The three statistical methods (SRM, ES, and paired t-test) that were utilized provided similar results in most instances. However, the external validity of assessing change was poor in the PROMIS PF and UE as well as some follow-up time points of the PROMIS PI and qDASH when mean scores were compared in the subsamples with no-change in condition versus meaningful change.

We tested a traditional time-frame for three-month and six-month follow-up capturing a window of 10 days on either side of the follow-up cut-off. Strict cut-off limits restrict the inclusion of patient scores for those who did not have follow-up visits that fit within the narrow time-frames. The relevance of the sampling cut-offs to the interpretation can be seen with the small sample size (18–20 participants) in the 6-month follow-up group (170–190 days). This restricted sample was the only time-point that resulted in a 95% CI around the effect size that ranged low enough to include potential for a small effect in the interpretation. In contrast, the larger sample sizes in the other follow-up periods resulted in CI’s with medium/large to large effects. We also tested 90 days and beyond and 180 days and beyond as alternative time-frames to test the robustness of these cut-offs to the measure’s responsiveness. Our study findings that comparable effect sizes could be seen across the differing follow-up cut-offs, with minimal exceptions, provides cross-validation for the use of commonly used three and six-month follow-up cut-off points.

It is interesting to note that the time-period in which change scores were the greatest differed for different instruments. For the PROMIS PF, there was little difference between change scores at 3 and 6 month follow-up. For the PROMIS PI, pain interference change was greater at the earlier follow-up points. The PROMIS UE and qDASH similarly showed more change in function at earlier time points. These differences likely represent the greater heterogeneity in patient condition and treatment factors that occur by later measurement periods, but may also reflect the nature of improvement in upper extremity disorders. It may also reflect the low level of functioning and high levels of pain reported by this sample of upper extremity patients at baseline visits.

Prior work on the measurement characteristics of the PROMIS UE, PF, and PI CAT in a hand and upper extremity patient population have demonstrated the validity of these measures while minimizing respondent burden [8, 29–32]. Whether or not these PROMIS instruments are able to detect patient reported change in health or function, however, has remained an important albeit open question. This study demonstrates the responsiveness of these three PROMIS instruments. Understanding responsiveness to change is essential in translational research to advance clinical trials, comparative effectiveness studies and most importantly, clinicians’ knowledge in interpreting outcome measures enabling more meaningful interactions with patients.

### Limitations

All patients visiting the hand and upper extremity orthopaedic clinic were included in the assessment of responsiveness, and we did not characterize our results based on individual diagnosis or treatments. Differing disease conditions and/or treatments may show different responsiveness indices, and therefore the findings of this study should be considered preliminary. Future work may include investigation of the responsiveness of the PROMIS instruments for individual conditions and treatments. The sample size for the 6-month follow-up was small and results from this time-point may not be as reliable as those with larger samples. We are continuing to collect data from patients and will conduct further study with larger samples and different time frames as data become available. Future work should be performed to analyze upper extremity conditions at varying levels of function, not just change, to see if instruments are as responsive to those with high functioning as to those with lower levels of function. It would also be useful to consider the differences by anchor score, of those reporting varying levels of improvement. The PROMIS PF has been shown to have a ceiling effect especially in relation to items that fall in the upper extremity areas of function. [29, 33] In this patient population, functioning levels were low, so the ceiling effect likely did not impact the results. Both the PROMIS PF and PROMIS UE would benefit from this additional analysis of responsiveness at the upper levels of function in future research, potentially using Rasch modeling based on the distribution of scores rather than the external anchoring.

## Conclusions

The PROMIS UE CAT, PF CAT, PI CAT, and qDASH were able to effectively detect change in physical function and pain interference in an orthopaedic hand and upper extremity clinic. The responsiveness of the PROMIS instruments demonstrated by this study adds to the prior rigorous psychometric validation of instruments reported in the literature, and should assist clinicians and researchers to make informed decisions regarding instrument selection in assessing patient reported outcomes in the upper extremity [34].

## Appendix

**Table 5:** PROMIS v1.2 Physical Function item bank

Item	ID	Questions
1	PFA10	Are you able to stand for one hour?^a^
2	PFA11	Are you able to do chores such as vacuuming or yard work?
3	PFA12	Are you able to push open a heavy door?
4	PFA13	Are you able to exercise for an hour?
5	PFA14r1	Are you able to carry a heavy object (over 10 pounds/ 5 kg)?
6	PFA15	Are you able to stand up from an armless straight chair?
7	PFA16r1	Are you able to dress yourself, including tying shoelaces and buttoning up your clothes?
8	PFA17	Are you able to reach into a high cupboard?
9	PFA18	Are you able to use a hammer to pound a nail?
10	PFA19r1	Are you able to run or jog for two miles (3 km)?
11	PFA20	Are you able to cut your food using eating utensils?
12	PFA21	Are you able to go up and down stairs at a normal pace?
13	PFA22	Are you able to open previously opened jars?
14	PFA23	Are you able to go for a walk of at least 15 min?
15	PFA25	Are you able to do yard work like raking leaves, weeding, or pushing a lawn mower?
16	PFA28	Are you able to open a can with a hand can opener?
17	PFA29r1	Are you able to pull heavy objects (10 pounds/5 kg) towards yourself?
18	PFA30	Are you able to step up and down curbs?
19	PFA31r1	Are you able to get up from the floor from lying on your back without help?
20	PFA32	Are you able to stand with your knees straight?
21	PFA33	Are you able to exercise hard for half an hour?
22	PFA34	Are you able to wash your back?
23	PFA35	Are you able to open and close a zipper?
24	PFA36	Are you able to put on and take off a coat or jacket?
25	PFA37	Are you able to stand for short periods of time?
26	PFA38	Are you able to dry your back with a towel?
27	PFA39r1	Are you able to run at a fast pace for two miles (3 km)?
28	PFA40	Are you able to turn a key in a lock?
29	PFA41	Are you able to squat and get up?
30	PFA42	Are you able to carry a laundry basket up a flight of stairs?
31	PFA43	Are you able to write with a pen or pencil?
32	PFA44	Are you able to put on a shirt or blouse?
33	PFA45	Are you able to get out of bed into a chair?
34	PFA47	Are you able to pull on trousers?
35	PFA48	Are you able to peel fruit?
36	PFA49	Are you able to bend or twist your back?
37	PFA50	Are you able to brush your teeth?
38	PFA51	Are you able to sit on the edge of a bed?
39	PFA52	Are you able to tie your shoelaces?
40	PFA53	Are you able to run errands and shop?
41	PFA54	Are you able to button your shirt?
42	PFA55	Are you able to wash and dry your body?
43	PFA56	Are you able to get in and out of a car?
44	PFA8	Are you able to move a chair from one room to another?
45	PFA9	Are you able to bend down and pick up clothing from the floor?
46	PFB10	Are you able to climb up five steps?
47	PFB11	Are you able to wash dishes, pots, and utensils by hand while standing at a sink?
48	PFB12	Are you able to make a bed, including spreading and tucking in bed sheets?
49	PFB13	Are you able to carry a shopping bag or briefcase?
50	PFB14	Are you able to take a tub bath?
51	PFB15	Are you able to change the bulb in a table lamp?
52	PFB16	Are you able to press with your index finger (for example ringing a doorbell)?
53	PFB17	Are you able to put on and take off your socks?
54	PFB18	Are you able to shave your face or apply makeup?
55	PFB19	Are you able to squeeze a new tube of toothpaste?
56	PFB20	Are you able to cut a piece of paper with scissors?
57	PFB21	Are you able to pick up coins from a table top?
58	PFB22	Are you able to hold a plate full of food?
59	PFB23	Are you able to pour liquid from a bottle into a glass?
60	PFB24	Are you able to run a short distance, such as to catch a bus?
61	PFB25	Are you able to push open a door after turning the knob?
62	PFB26	Are you able to shampoo your hair?
63	PFB27	Are you able to tie a knot or a bow?
64	PFB28r1	Are you able to lift 10 pounds (5 kg) above your shoulder?
65	PFB29	Are you able to lift a full cup or glass to your mouth?
66	PFB30	Are you able to open a new milk carton?
67	PFB31	Are you able to open car doors?
68	PFB32	Are you able to stand unsupported for 10 min?
69	PFB33	Are you able to remove something from your back pocket?
70	PFB34	Are you able to change a light bulb overhead?
71	PFB36	Are you able to put on a pullover sweater?
72	PFB37	Are you able to turn faucets on and off?
73	PFB39r1	Are you able to reach and get down a 5 pound (2 kg) object from above your head?
74	PFB40	Are you able to stand up on tiptoes?
75	PFB41	Are you able to trim your fingernails?
76	PFB42	Are you able to stand unsupported for 30 min?
77	PFB56r1	Are you able to lift one pound (0.5 kg) to shoulder level without bending your elbow?
78	PFB8r1	Are you able to carry two bags filled with groceries 100 yards (100 m)?
79	PFB9	Are you able to jump up and down?
80	PFC13r1	Are you able to run 100 yards (100 m)?
81	PFC29	Are you able to walk up and down two steps?
82	PFC31	Are you able to reach into a low cupboard?
83	PFC32	Are you able to climb up 5 flights of stairs?
84	PFC33r1	Are you able to run ten miles (16 km)?
85	PFC38	Are you able to walk at a normal speed?
86	PFC39	Are you able to stand without losing your balance for several minutes?
87	PFC40	Are you able to kneel on the floor?
88	PFC41	Are you able to sit down in and stand up from a low, soft couch?
89	PFC43	Are you able to use your hands, such as for turning faucets, using kitchen gadgets, or sewing?
90	PFC45r1	Are you able to sit on and get up from the toilet?
91	PFC46	Are you able to transfer from a bed to a chair and back?
92	PFC47	Are you able to be out of bed most of the day?
93	PFC49	Are you able to water a house plant?
94	PFC51	Are you able to wipe yourself after using the toilet?
95	PFC52	Are you able to turn from side to side in bed?
96	PFC53	Are you able to get in and out of bed?
97	PFC6r1	Are you able to walk a block (100 m) on flat ground?
98	PFC7r1	Are you able to run five miles (8 km)?
99	PFA1	Does your health now limit you in doing vigorous activities, such as running, lifting heavy objects, participating in strenuous sports?^b^
100	PFA3	Does your health now limit you in bending, kneeling, or stooping?
101	PFA4	Does your health now limit you in doing heavy work around the house like scrubbing floors, or lifting or moving heavy furniture?
102	PFA5	Does your health now limit you in lifting or carrying groceries?
103	PFA6	Does your health now limit you in bathing or dressing yourself?
104	PFB1	Does your health now limit you in doing moderate work around the house like vacuuming, sweeping floors or carrying in groceries?
105	PFB3	Does your health now limit you in putting a trash bag outside?
106	PFB43	Does your health now limit you in taking care of your personal needs (dress, comb hair, toilet, eat, bathe)?
107	PFB44	Does your health now limit you in doing moderate activities, such as moving a table, pushing a vacuum cleaner, bowling, or playing golf?
108	PFB48	Does your health now limit you in taking a shower?
109	PFB49	Does your health now limit you in going for a short walk (less than 15 min)?
110	PFB5r1	Does your health now limit you in hiking a couple of miles (3 km) on uneven surfaces, including hills?
111	PFB51	Does your health now limit you in participating in active sports such as swimming, tennis, or basketball?
112	PFB54	Does your health now limit you in going OUTSIDE the home, for example to shop or visit a doctor’s office?
113	PFB7	Does your health now limit you in doing strenuous activities such as backpacking, skiing, playing tennis, bicycling or jogging?
114	PFC10	Does your health now limit you in climbing several flights of stairs?
115	PFC12	Does your health now limit you in doing two hours of physical labor?
116	PFC35	Does your health now limit you in doing eight hours of physical labor?
117	PFC36r1	Does your health now limit you in walking more than a mile (1.6 km)?
118	PFC37	Does your health now limit you in climbing one flight of stairs?
119	PFC54	Does your health now limit you in getting in and out of the bathtub?
120	PFC56	Does your health now limit you in walking about the house?
121	PFB50	How much difficulty do you have doing your daily physical activities, because of your health?^c^

^a^Response options for questions 1–98 are 1 = Unable to do; 2 = With much difficulty; 3 = With some difficulty; 4 = With a little difficulty; 5 = Without any difficulty

^b^Response options for questions 99–123 are 1 = Cannot do; 2 = Quite a lot; 3 = Somewhat; 4 = Very little; 5 = Not at all

^c^Response options for question 121 are 1 = Can’t do because of health, 2 = A lot of difficulty; 3 = Some difficulty; 4 = A little bit of difficulty; 5 = No difficulty at all

**Table 6:** PROMIS v1.2 Upper Extremity item bank

Item	ID	Question
1	PFA16r1	Are you able to dress yourself, including tying shoelaces and buttoning your clothes?
2	PFA17	Are you able to reach into a high cupboard?
3	PFA18	Are you able to use a hammer to pound a nail?
4	PFA20	Are you able to cut your food using eating utensils?
5	PFA28	Are you able to open a can with a hand can opener?
6	PFA29r1	Are you able to pull heavy objects (10 pounds/5 kg) towards yourself?
7	PFA35	Are you able to open and close a zipper?
8	PFA38	Are you able to dry your back with a towel?
9	PFA44	Are you able to put on a shirt or blouse?
10	PFA48	Are you able to peel fruit?
11	PFA54	Are you able to button your shirt?
12	PFB21	Are you able to pick up coins from a table top?
13	PFB22	Are you able to hold a plate full of food?
14	PFB30	Are you able to open a new milk carton?
15	PFB33	Are you able to remove something from your back pocket?
16	PFB36	Are you able to put on a pullover sweater?

Response options for questions 1–16 are 1 = Unable to do; 2 = With much difficulty; 3 = With some difficulty; 4 = With a little difficulty; or 5 = Without any difficulty

**Table 7:** PROMIS v1.1 Pain Interference item bank

Item	ID	Question
1	PAININ1	In the past 7 days, how difficult was it for you to take in new information because of pain?^a^
2	PAININ3	In the past 7 days, how much did pain interfere with your enjoyment of life?^a^
3	PAININ5	In the past 7 days, how much did pain interfere with your ability to participate in leisure activities?^a^
4	PAININ6	In the past 7 days, how much did pain interfere with your close personal relationships?^a^
5	PAININ8	In the past 7 days, how much did pain interfere with your ability to concentrate?^a^
6	PAININ9	In the past 7 days, how much did pain interfere with your day to day activities?^a^
7	PAININ10	In the past 7 days, how much did pain interfere with your enjoyment of recreational activities?^a^
8	PAININ11	In the past 7 days, how often did you feel emotionally tense because of your pain?^a^
9	PAININ12	In the past 7 days, how much did pain interfere with the things you usually do for fun?^a^
10	PAININ13	In the past 7 days, how much did pain interfere with your family life?^a^
11	PAININ17	In the past 7 days, how much did pain interfere with your relationships with other people?^a^
12	PAININ18	In the past 7 days, how much did pain interfere with your ability to work (included work at home)?^a^
13	PAININ19	In the past 7 days, how much did pain make it difficult to fall asleep?^a^
14	PAININ20	In the past 7 days, how much did pain feel like a burden to you?^a^
15	PAININ22	In the past 7 days, how much did pain interfere with work around the home?^a^
16	PAININ31	In the past 7 days, how much did pain interfere with your ability to participate in social activities? ^a^
17	PAININ34	In the past 7 days, how much did pain interfere with your household chores? ^a^
18	PAININ35	In the past 7 days, how much did pain interfere with your ability to make trips from home that kept you gone for more than 2 h? ^a^
19	PAININ36	In the past 7 days, how much did pain interfere with your enjoyment of social activities? ^a^
20	PAININ48	In the past 7 days, how much did pain interfere with your ability to do household chores? ^a^
21	PAININ49	In the past 7 days, how much did pain interfere with your ability to remember things? ^a^
22	PAININ56	In the past 7 days, how irritable did you feel because of pain? ^a^
23	PAININ14	In the past 7 days, how much did pain interfere with doing your tasks away from home (e.g., getting groceries, running errands)? ^a^
24	PAININ16	In the past 7 days, how often did pain make you feel depressed?^b^
25	PAININ24	In the past 7 days, how often was pain distressing to you? ^b^
26	PAININ26	In the past 7 days, how often did pain keep you from socializing with others?^b^
27	PAININ29	In the past 7 days, how often was your pain so severe you could think of nothing else? ^b^
28	PAININ32	In the past 7 days, how often did pain make you feel discouraged?^b^
29	PAININ37	In the past 7 days, how often did pain make you feel anxious?^b^
30	PAININ38	In the past 7 days, how often did you avoid social activities because it might make you hurt more?^b^
31	PAININ40	In the past 7 days, how often did pain prevent you from walking more than 1 mile?
32	PAININ42	In the past 7 days, how often did pain prevent you from standing for more than one hour?^b^
33	PAININ46	In the past 7 days, how often did pain make it difficult for you to plan social activities? ^b^
34	PAININ47	In the past 7 days, how often did pain prevent you from standing for more than 30 min?^b^
35	PAININ50	In the past 7 days, how often did pain prevent you from sitting for more than 30 min?^b^
36	PAININ51	In the past 7 days, how often did pain prevent you from sitting for more than 10 min?^b^
37	PAININ52	In the past 7 days, how often was it hard to plan social activities because you didn’t know if you would be in pain?^b^
38	PAININ53	In the past 7 days, how often did pain restrict your social life to your home?^b^
39	PAININ55	In the past 7 days, how often did pain prevent you from sitting for more than one hour? ^b^
40	PAININ54	In the past 7 days, how often did pain keep you from getting into a standing position?^b^

^a^Response options for questions 1–23 are 1 = Not at all; 2 = A little bit; 3 = Somewhat; 4 = Quite a bit; 5 = Very Much

^b^Response options for questions 24–40 are 1 = Never; 2 = Rarely; 3 = Sometimes; 4 = Often; 5 = Always

**Table 8:** qDASH item bank

Item	Question
	*Please rate your ability to do the following activities in the last week.*
1	Open a tight or new jar.^a^
2	Do heavy household chores (e.g., wash walls, floors).^a^
3	Carry a shopping bag or briefcase.^a^
4	Wash your back.^a^
5	Use a knife to cut food.^a^
6	Recreational activities in which you take some force or impact through your arm, shoulder or hand (e.g., golf, hammering, tennis, etc.).^a^
7	During the past week, to what extent has your arm, shoulder or hand problem interfered with your normal social activities with family, friends, neighbors or groups?^b^
8	During the past week, were you limited in your work or other regular daily activities as a result of your arm, shoulder or hand problem?^c^
	*Please rate the severity of the following symptoms in the last week.*
9	Arm, shoulder or hand pain.^d^
10	Tingling (pins and needles) in your arm, shoulder or hand.^d^
11	During the past week, how much difficulty have you had sleeping because of the pain in your arm, shoulder or hand?^a^

^a^Response options for questions 1–6, 11 are 1 = No difficulty; 2 = Mild difficulty; 3 = Moderate difficulty; 4 = Severe difficulty; 5 = So much difficulty that I can’t sleep

^b^Response options for question 7 are 1 = Not at all; 2 = Slightly; 3 = Moderately; 4 = Quite a bit; 5 = Extremely

^c^Response options for question 8 are 1 = Not limited at all; 2 = Slightly limited; 3 = Moderately limited; 4 = Very limited; 5 = Unable

^d^Response options for questions 9–10 are 1 = None; 2 = Mild; 3 = Moderate; 4 = Severe; 5 = Extreme

## Abbreviations

ES: Effect size
PF CAT: PROMIS Physical Function computerized adaptive test
PI CAT: PROMIS Pain Interference computerized adaptive test
PRO: Patient-reported outcome
PROMIS: Patient Reported Outcomes Measurement Information System
qDASH: Disabilities of the Arm, Shoulder, and Hand; shortened version
SRM: Standardized response mean
UE CAT: PROMIS Upper Extremity computerized adaptive test
